# LncRNA NR_003923 promotes cell proliferation, migration, fibrosis, and autophagy via the miR-760/miR-215-3p/IL22RA1 axis in human Tenon’s capsule fibroblasts

**DOI:** 10.1038/s41419-019-1829-1

**Published:** 2019-08-07

**Authors:** Yang Zhao, Feng Zhang, Zheng Pan, Haomin Luo, Ke Liu, Xuanchu Duan

**Affiliations:** 1 Department of Ophthalmology, The Second Xiangya Hospital, Central South University, Changsha, Hunan Province China; 2 Changsha Aier Eye Hospital, Aier Glaucoma Research Institute, Aier School of Ophthalmology, Central South University, Changsha, Hunan Province China

**Keywords:** Chaperone-mediated autophagy, Cell invasion

## Abstract

Noncoding RNAs (ncRNAs), including long ncRNAs (lncRNA) have manifested an important role in the pathophysiology of many diseases. Glaucoma is a primary cause of irreversible blindness worldwide. However, the involvement of lncRNAs in glaucoma remains largely unknown. Here, we performed the lncRNA expression assay based on clinical tissues and identified a specific functional lncRNA, NR_003923, and investigated its potential role in glaucoma. Knockdown of NR_003923 in human Tenon’s capsule fibroblast cells (HTFs) inhibited TGF-β-induced cell migration, proliferation, fibrosis, and autophagy. The dual luciferase reporter assay confirmed that miR-760 and miR-215-3p interacted with NR_003923. miR-760 and miR-215-3p inhibitor reversed the effects of NR_003923 and TGF-β-induced cell apoptosis. Moreover, the expression of miR-760 and miR-215-3p was decreased in glaucoma comparing with control. Furthermore, through microarray we found IL22RA1 was increased in glaucoma and both of miR-760 and miR-215-3p bound to the 3′ UTR of IL22RA1. Overexpression of IL22RA1 enhanced HTFs migration and proliferation, while miR-760 and miR-215-3p mimics reversed these promotive biological roles induced by IL22RA1. In conclusion, NR_003923 and IL22RA1 might contribute to glaucoma progression and be a novel and potential biomarkers and therapeutic targets for glaucoma.

## Introduction

Glaucoma is a severe eye disease and a major cause of irreversible blindness throughout the world. Glaucoma is characterized by the irreversible apoptosis of retinal ganglion cells and visual field loss^1^. Filtering surgery is currently regarded as the most effective therapy for glaucoma, excessive scarring due to filtering surgery is the most common reason for an unsuccessful surgical treatment^2^. Accumulating evidence has indicated that glaucoma are associated with cell proliferation, fibrosis, migration, and autophagy, which induced by wound healing after surgery^3^. Proliferation of Tenon’s capsule fibroblasts (HTFs) has been demonstrated as a key role in conjunctival repair and scarring^4^. HTFs become activated to promote wound-healing processes such as cell proliferation and migration, the synthesis of extracellular matrix components (ECM)^1^, and autophagy^5^. Phenotype transition from fibroblasts to myofibroblasts has demonstrated as a key proof of subconjunctival fibrosis^5^.

Transforming growth factor β (TGF-β) shown to play a crucial role in excessive subconjunctival fibrosis caused failure after glaucoma-filtering surgery and as a stimulator in cell proliferation, migration, and autophagy processes in an in vivo rabbit model and HTFs in vivo^6–9^. However, the underlying mechanism of physiological and pathological contexts associated with glaucoma or the TGF-β1-induced cell model still need to be fully understood.

Genome-sequencing projects have discovered that <2% of genes in the total human genome code for proteins, and the remainder (>90%) are transcribed as noncoding RNAs (ncRNAs)^10^. Among those ncRNAs, long noncoding RNAs (lncRNAs), (ncRNAs > 200 nucleotides in length), have long been regarded as junk RNAs. Recently, however, lncRNAs have been shown to play various roles in the development of diseases, including glaucoma^11^. Previous study suggested that the interaction between lncRNA MEG3 and Nrf2 plays as a key role in mediating the proliferation of HTFs^12^. Specifically, lncRNA MEG3 appears to inhibit HTFs proliferation by directly binding to Nrf2^12^. Another LncRNA, LncRNA MALAT1 inhibits retinal ganglion cell apoptosis via the activation PI3K/AKT signaling pathway in the chronic high-intraocular pressure (IOP) rat model of glaucoma^13^. Little doubt now exists that lncRNAs play a crucial role in maintaining the homeostasis of a microenvironment. However, the underlying mechanism by which lncRNAs affect the occurrence and development of glaucoma remains unclear.

In this study, we performed microarray assays to obtain an overview of the expression profiles of various lncRNAs and mRNAs in glaucoma and normal tissues. We found that two miRNAs (miR-760 and miR-215) were downregulated in glaucoma tissues had been reported to be associated with the status of both oral submucous fibrosis and cancer fibrosis^14,15^. Furthermore, a target predication study identified interleukin-22 receptor alpha-1 (IL22RA1) as a target of both miR-760 and miR-215. IL22 induction induced both IL22RA1 and FN1 expression in pancreatic stellate cells^16^, indicating its protective role in pancreatic fibrosis. Lastly, a novel lncRNA (lncRNA NR_003923) that is transcribed by the gene that encodes the guanylyl cyclase subunit beta 2 (GUCY1B2), a member of the soluble guanylate cyclase family that plays crucial roles in glaucoma^17^, was identified from a list of differentially expressed lncRNAs (DE-lncRNAs) as possibly being capable of interacting with miR-760 or miR-215. Accordingly, we speculated that such an interaction or the NR_003923-miR-760/miR-325-3p-IL22RA1 axis might play an important role in mediating HTFs proliferation, migration, fibrosis, and autophagy. This study was performed to investigate the role played by this axis in glaucoma, and our results provide novel information that can be used in future investigations of glaucoma.

## Results

### Analysis of differentially expressed lncRNAs in glaucoma and control tissues

**Fig. 1:**
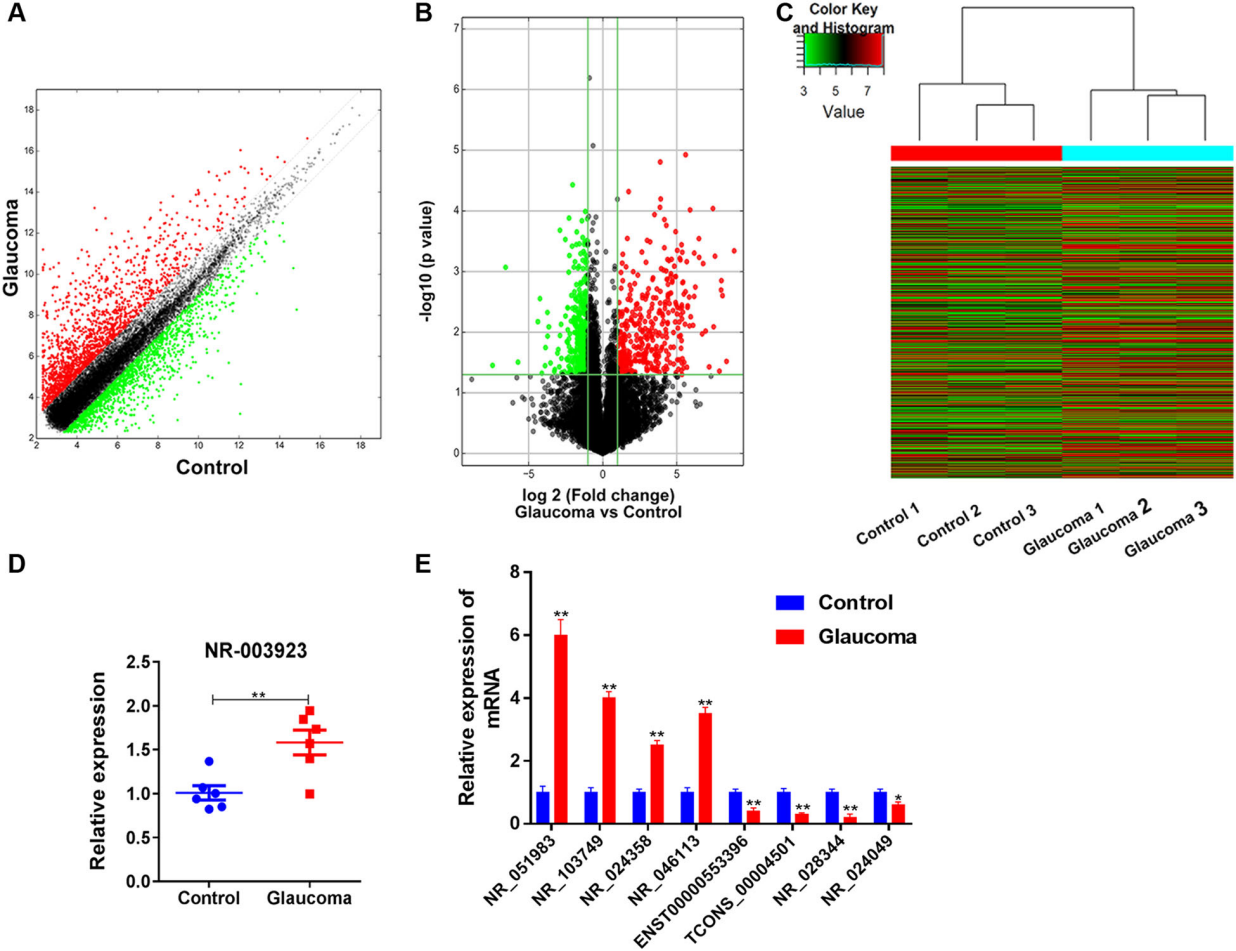
The lncRNA expression profiles in normal and glaucoma tissues were compared, and NR_003923 was selected for further investigation. **a** A scatter-plot was created to assess the lncRNA expression profiles. **b** A volcano plot was used to identify lncRNAs that showed a ≥twofold change in expression with a *p*-value ≤ 0.05. **c** The hierarchical clustering of lncRNAs according to our filtering criteria. **d** The difference in NR_003923 expression between control and glaucoma tissues. The levels of NR_003923 expression were significantly higher in glaucoma tissues. **e** The expression of eight randomly selected lncRNAs. **p < 0.01 vs control

In an attempt to obtain an overview of the lncRNAs that are differentially expressed in glaucoma tissues comparing with normal tissues, we collected tissue specimens from glaucoma patients and analyzed by using microarray methods. Our lncRNA expression profiles showed that 3093 lncRNAs were differentially expressed in glaucoma comparing with normal tissues, 1638 of the DE-lncRNAs were upregulated in the glaucoma tissues, and 1455 were downregulated (Fig. 1a). Among the DE-lnRNAs, 705 showed a >twofold change in expression in glaucoma tissues and 768 showed a lesser but still significant degree of change in the glaucoma tissues (*p* < 0.05) (Fig. 1b). Hierarchical-clustering results showed the distinguishing expression profiles of the 1475 DE-lncRNAs in glaucoma tissues and control tissues (Fig. 1c). Among the upregulated DE-LncRNAs, we first validated the expression of lncRNA NR_003923 by using real-time PCR. Our results indicated that NR_003923 levels were elevated in glaucoma tissues, and this finding was consistent with the results of our microarray assays (Fig. 1d). To strengthen the validation, we also determined the change in expression of other eight randomly selected lncRNAs in glaucoma tissues relative to their expression in the control tissues (Fig. 1e).

### NR_003923 promoted the TGF-β-induced proliferation, migration, and fibrosis of HTFs via the sponging of miR-760 and miR-215-3p

We used TGF-β-treated human tenon’s capsule fibroblasts to mimics the glaucoma model in vitro. As shown in Fig. S1, TGF-β significantly enhanced cell viability in a dose-dependent manner after incubation for 24 h and 48 h (Fig. S1, A). In addition, TGF-β at 1, 2, or 4 ng/mL promoted cell migration (Fig. S1, B, C) and significantly elevated expression of α-SMA and fibronectin (FN), and suppressed E-cadherin and β-catenin expression (Fig. S1, D–F).

**Fig. 2:**
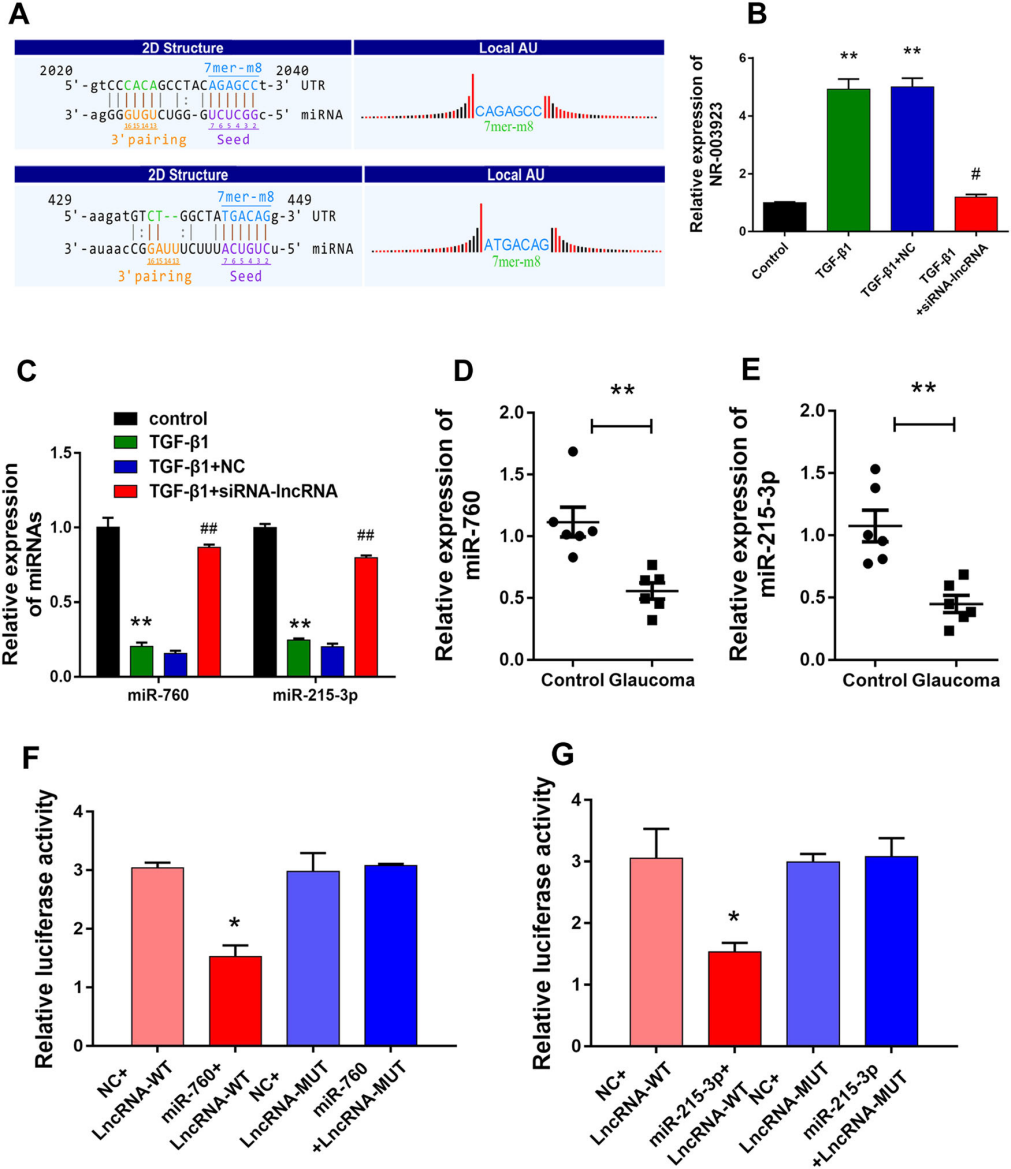
NR_003923 induced by TGF-β directly interacted with miR-760 and miR-215-3p. **a** MiR-760 and miR-215-3p were selected based on a ceRNA analysis. **b** NR_003923 was mediated by TGF-β. **c** MiR-760 and miR-215-3p expression displayed reversed trends when compared with NR_003923 expression. **d, e** MiR-760 and miR-215-3p were downregulated in glaucoma. *p < 0.05, **p < 0.01 vs control. **f, g** The dual luciferase reporter assay confirmed thatmiR-760 and miR-215-3p could directly bind with NR_03923. *p < 0.05 vs NC

To explored the role of NR_003923 in TGF-β-induced HTFs, the interactions between lncRNA NR_003923 and miRNAs were predicted by KangChen Bio-tech (Shanghai, China), DIANA database, Target Human Scan software (Version 7.2; http://www.targetscan.org/vert_72/), and lncRNA downstream target database of StarBase v2.0,25. Two miRNAs (miR-760 and miR-215-3p) with predicted binding sites for NR_003923 (Fig. 2a) were selected for investigation. A real-time PCR analysis showed that NR_003923 expression was increased in HTFs treated with TGF-β. However, the expression of NR_003923 was suppressed by siRNA-targeting NR_003923 (Fig. 2b). Interestingly, both miR-760 and miR-215-3p displayed an expression trend that was opposite of that displayed by NR_003923. Specifically, miR-760 and miR-215-3p expression were downregulated in TGF-β-treated cells, but upregulated in cells treated with NR_003923 siRNA (Fig. 2c). Therefore, we further investigated the expression of miR-760 and miR-215-3p in clinical tissues. As expected, both miR-760 and miR-215-3p were found to be downregulated in glaucoma tissues, when compared with their expression levels in control tissues (Fig. 2d, e). These results indicated that these two miRNAs might play a protective role in glaucoma.

**Fig. 3:**
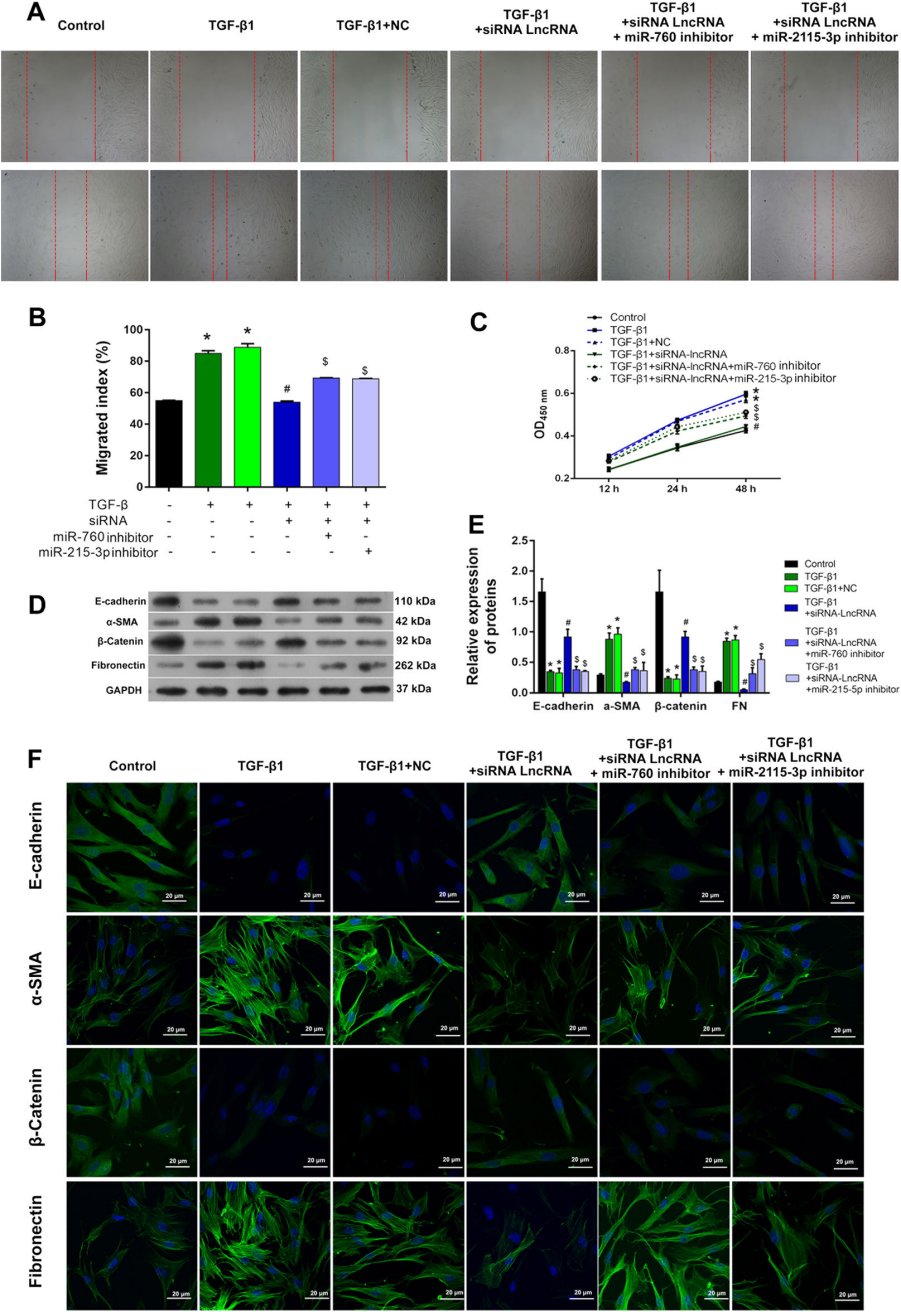
The effect of interaction of NR_003923 with miR-760 and miR-215-3p on the migration and proliferation of Tenon’s capsule fibroblasts and fibrosis-related proteins. **a, b** The miR-760/miR-215-3p inhibitor reversed the suppressive effect of siRNA NR_003923 on cell migration. **c** The miR-760/miR-215-3p inhibitor reversed the cell proliferation induced by siRNA NR_003923. **d–f** The expression of E-cadherin, α-SMA, β-catenin, and fibronectin in siRNA NR_003923 treated cells could be recovered by transfection with the miR-760/miR-215-3p inhibitor. *p < 0.05 vs control. ^#^*p* < 0.05 vs TGFβ + NC, ^$^*p* < 0.05 vs TGF-β + siRNA. Magnification, ×400

A dual luciferase reporter assay further verified the direct binding of NR_003923 with miR-760 and miR-215-3p (Fig. 2f, g). To further verify the role played by interactions between NR_003923 and miRNAs (miR-760 and miR-215-3p), we transfected miR-760 or miR-215-3p inhibitor into HTFs that had been transfected with NR_003923 siRNA. Transfection with siRNA-targeting NR_003923 significantly inhibited cell migration and proliferation of HTFs when compared with HTFs treated with TGF-β alone (Fig. 3). However, these inhibitory effects were reversed by transfection with the miR-760 or miR-215-3p inhibitor (Fig. 3a–c). Western blot and immunofluorescence assays showed that TGF-β significantly reduced the expression of E-cadherin and β-catenin and induced the expression of α-SMA and FN. NR_003923 siRNA significantly reversed these effects. Moreover, both of miR-760 and miR-215-3p inhibitor recovered the expression of fibrosis markers reduced by NR_003923 under TGF-β treatment (Fig. 3d–f). These results showed that NR_003923 promoted the TGF-β-induced proliferation, migration, and fibrosis of HTFs by sponging miR-760 and miR-215-3p.

### Interaction of NR_003923 with miR-760 and miR-215-3p-altered autophagy in HTFs

**Fig. 4:**
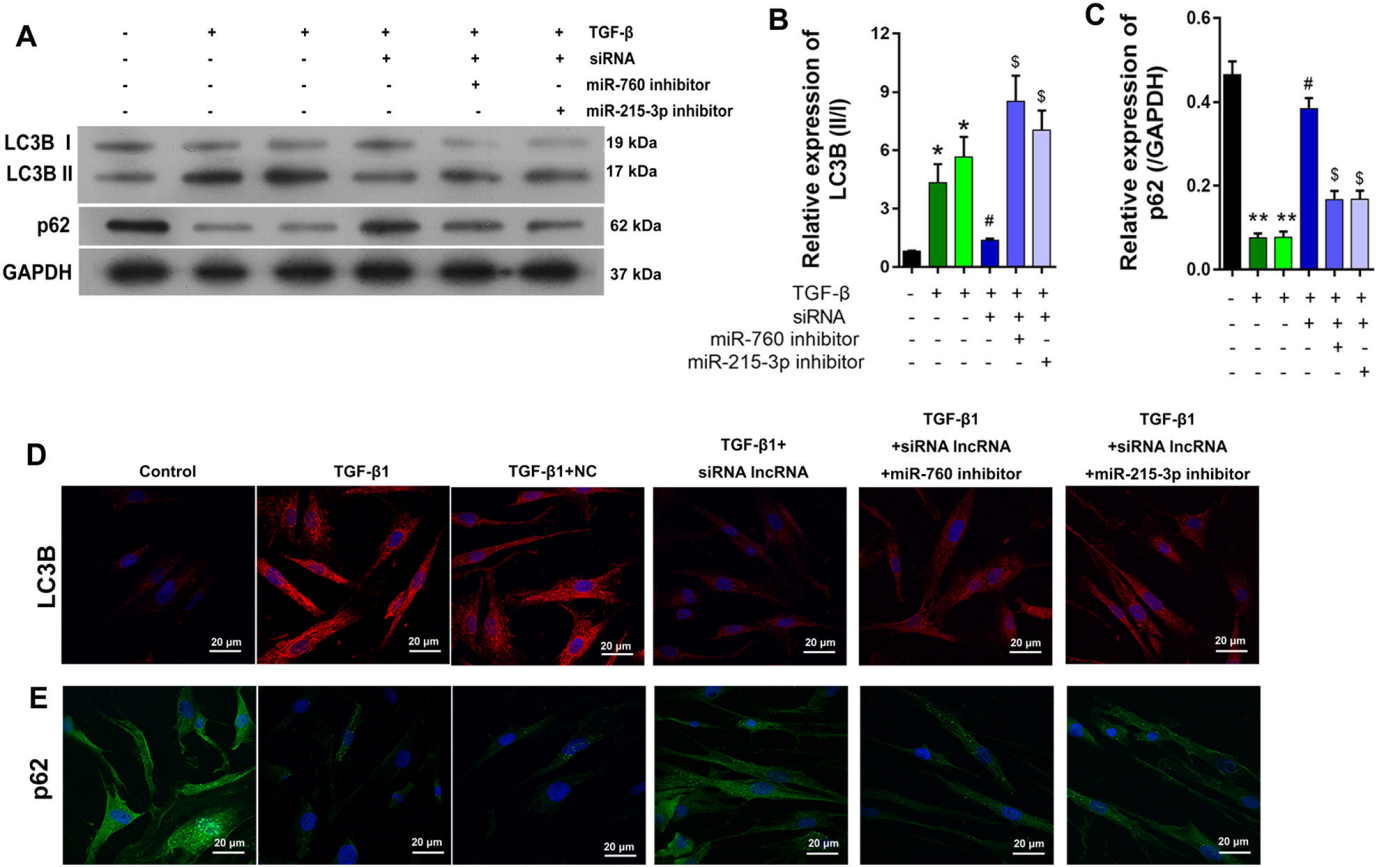
Autophagy in TGF-β-treated Tenon’s capsule fibroblasts was mediated by siRNA, miR-760, and miR-215-3p. **a–c** The miR-760/miR-215-3p inhibitor enhanced LC3BII expression (**b**) and suppressed p62 expression (**c**) in siRNA transfected Tenon’s capsule fibroblasts, as based on the effects of TGF-β incubation. *p < 0.05, **p < 0.01 vs control, #*p* < 0.05 vs TGFβ + NC, ^$^*p* < 0.05 vs TGF-β + siRNA. **d** LC3B and p62 expression were measured with immunofluorescence assays. Magnification, ×400

To explore how the interaction of NR_003923 with miR-760 and miR-215-3p-affected autophagy in HTFs, Western blot and real-time PCR studies were performed to detect the expression of autophagy-related proteins. As shown in Fig. 4, the LC3BII/I ratios were significantly higher in TGF-β-treated cells, however, these ratios were decreased by transfection with NR_003923 siRNA, and increased by transfection with the miR-760 or miR-215-3p inhibitor (Fig. 4a, b). As a substrate for autophagy, p62 displayed an expression tendency that was totally opposite of that displayed by the LC3BII/I ratio (Fig. 4a, c). Furthermore, the results of immunofluorescence assays were similar to those of western blot assays (Fig. 4d). These results indicated that inhibition of NR_003923 suppressed TGF-β induced autophagy, while miR-760 and miR-215-3p, the target of NR_003929, reversed these effects.

### MiR-760 and miR-215-3p targeted the 3′UTR of IL22RA1

**Fig. 5:**
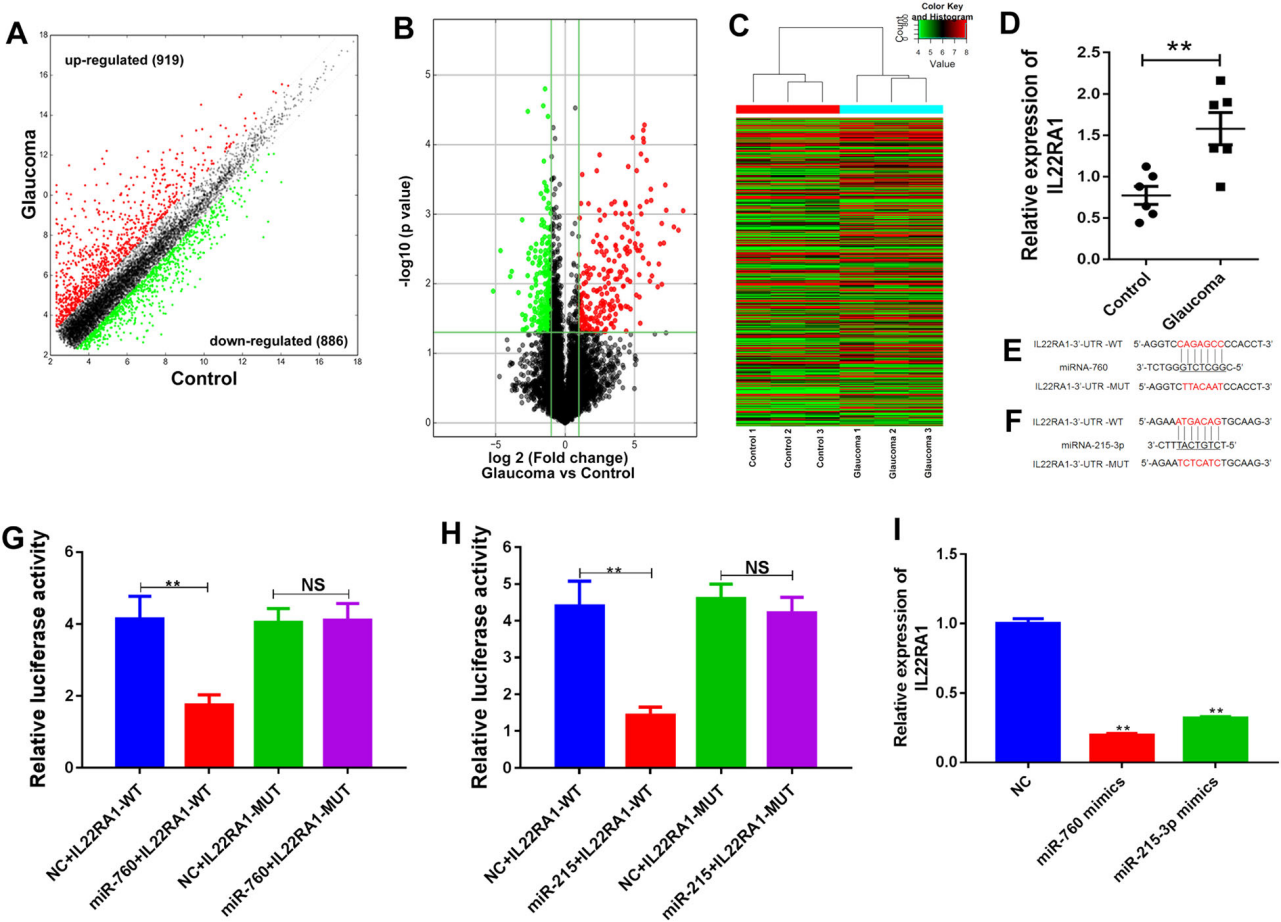
MiR-760 and miR-215-3p bound to the 3′UTR of IL22RA1 and inhibited IL22RA1 expression. **a** A scatter-plot was used to assess the expression profiles of mRNAs. **b** Volcano plots were used to identify mRNAs with a ≥ twofold change in expression and a *p*-value ≤ 0.05. **c** The hierarchical clustering of mRNAs according to our filtering criteria. **d** The difference in IL22RA1 expression between control and glaucoma tissues. IL22RA1 was expressed at significantly higher levels in glaucoma tissues. **p < 0.01 vs control. **e, f** Mutation sequences were introduced into the binding site for miR-760 and miR-215-3p in the 3′UTR of IL22RA1 mRNA. **g, h** Dual luciferase reporter assays were used to demonstrate that miR-760 and miR-215-3p could bind to the 3′UTR of IL22RA1 mRNA. **I** IL22RA1 expression was inhibited by the miR-760 and miR-215-3p mimics. **p < 0.01 vs NC

To obtain an overview of the interactions that occur between lncRNAs and mRNAs that were differentially expressed in glaucoma comparing with normal tissues, we performed a microarray investigation from three pairs of tissues. As shown in Fig. 5, among a total of 1805 mRNAs that were expressed, 929 were upregulated and 886 were downregulated in the glaucoma tissues (Fig. 5a). Among the mRNAs that were differentially expressed, 219 were significantly upregulated and 255 were significantly downregulated in the glaucoma tissues when compared with the normal tissues (*p* < 0.05, |log_2_(FC)| ≥ 1) (Fig. 5b). A hierarchical-clustering analysis showed the distinct expression patterns of the 474 genes that showed significant levels of differential expression (Fig. 5c). Among those genes, *IL22RA1* was predicated to be a target of both miR-760 and miR-215-3p, and its expression was upregulated in the glaucoma tissues when compared with the control tissues (Fig. 5d–f). Dual luciferase report assays further verified the interaction between miR-760 or miR-215-3p and IL22RA1 mRNA. (Fig. 5g, h). In addition, a significant downregulation of IL22RA1 expression was detected in cells transfected with miR-760 or miR-215-3p mimics (Fig. 5i). These results suggest that miR-760 and miR-215-3p might mediate cellular functions in HTFs by regulating IL22RA1 expression, which might play a promotive role in glaucoma.

### MiR-760 and miR-215-3p rescued the promoted effect of IL22RA1 on HTF proliferation, migration, fibrosis, and autophagy

**Fig. 6:**
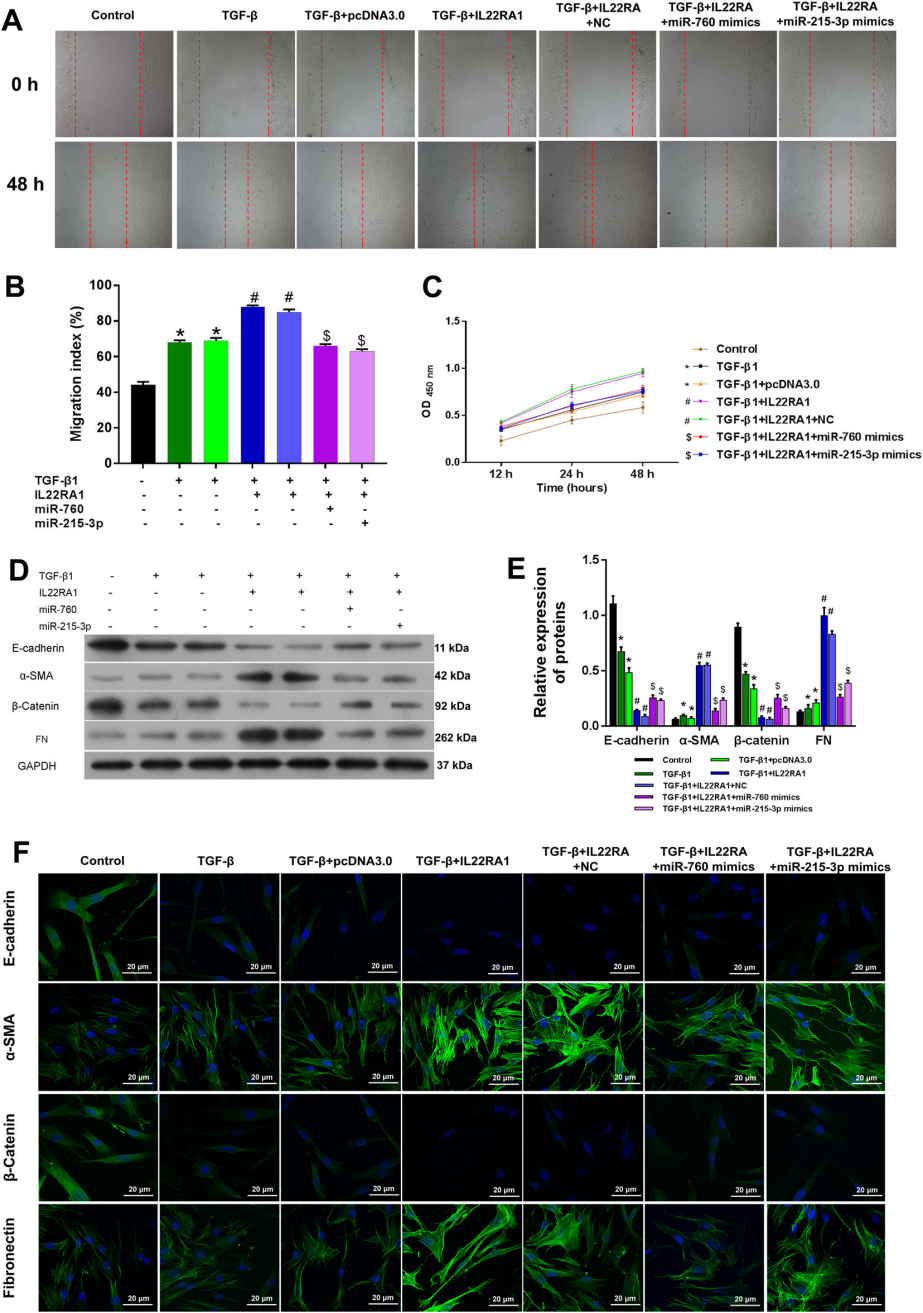
The effect of interaction of IL22RA1 with miR-760 and miR-215-3p on the migration and proliferation of Tenon’s capsule fibroblasts and fibrosis-related proteins. **a, b** MiR-760 and miR-215-3p reversed the increase in cell migration induced by IL22RA1. **c** MiR-760 and miR-215-3p reversed the cell proliferation induced by IL22RA1. *p < 0.05 vs control. ^#^*p* < 0.05 vs TGFβ + NC, ^$^*p* < 0.05 vs TGF-β + IL22RA1. **d**–**f** Expression of E-cadherin, α-SMA, β-catenin, and fibronectin in IL22RA1 treated cells were recovered by transfection of miR-760 and miR-215-3p. *p < 0.05 vs control. ^#^*p* < 0.05 vs TGFβ + NC, ^$^*p* < 0.05 vs TGF-β + IL22RA1. Magnification, ×400

Next we transfected miR-760 or miR-215-3p into HTFs that been previously transfected with *IL22RA1*. As shown in Fig. 6, overexpression of IL22RA1 strongly promoted the migration and proliferation of HTFs (Fig. 6a–c). However, transfection with miR-760 or miR-215-3p mimics significantly attenuated the promotive effect caused by IL22RA1 overexpression.

Western blot and immunofluorescence assays were performed to assess the effects of IL22RA1, miR-760andmiR-215-3p on E-cadherin, α-SMA, β-catenin, and FN expression. The results confirmed that *IL22RA1* overexpression produced a synergistic effect on TGF-β-suppressed E-cadherin and β-catenin and TGF-β-elevated α-SMA and FN in HTFs (Fig. 6d–f). In contrast, transfection with the miR-760 or miR-215-3p mimics reversed the enhancive effect of IL22RA1 (Fig. 6d–f).

**Fig. 7:**
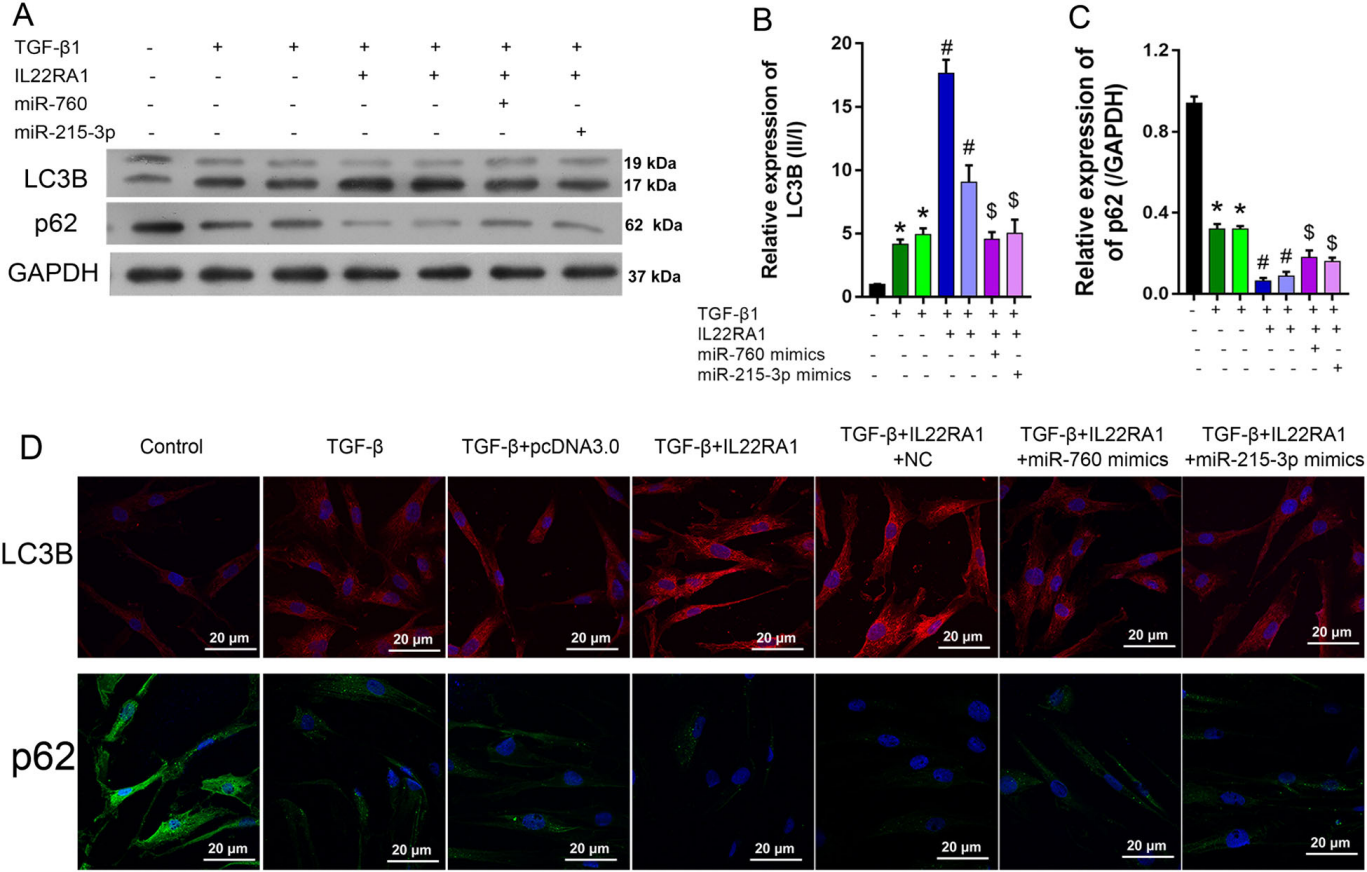
Autophagy in TGF-β-treated Tenon’s capsule fibroblasts was mediated by IL22RA1, miR-760, and miR-215-3p. **a**–**c** MiR-760 and miR-215-3p suppressed LC3BII expression (**b**) and promoted p62 expression (**c**) in IL22RA1-overexpressing Tenon’s capsule fibroblasts, as based on the effects of TGF-β incubation. *p < 0.05 vs control, ^#^*p* < 0.05 vs TGFβ + NC, ^$^*p* < 0.05 vs TGF-β + IL22RA1. **d** LC3B and p62 expression were measured with immunofluorescence assays. Magnification, ×400

Finally, we found that IL22RA1 overexpression resulted in a higher LC3II/I ratio and a lower level of p62 expression when compared with the effects produced by TGF-β treatment alone (Fig. 7a–c). Similar results were obtained with immunofluorescence assays (Fig. 7d). In contrast, transfection with the miR-760 or miR-215-3p mimics reversed the phenotype produced by IL22RA1 overexpression.

## Discussion

The activation and proliferation of HTFs in response to TGF-β stimulation is the main cause of surgical failure of glaucoma filtration surgery^11^. Presently, although surgery or drugs are often used to control IOP, patients with controlled IOP still suffer from filtration surgery failure due to excessive scar healing. Therefore, there is an urgent need to identify the underlying mechanism of excessive scar healing and identify therapies that inhibit HTFs proliferation, migration, fibrosis, and autophagy^9,18^. In the present study, we investigated a particular lncRNA might be involved in glaucoma, and also the interaction network of that lncRNA, with the hope that such information will contribute to finding a more effective treatment for glaucoma.

In general, the wound-healing process after a conjunctiva injury occurs in four phases: a hemostasis phase, inflammatory phase, proliferative phase, and a remodeling phase. Cytokines participate in all four phases, and a previous study showed that one cytokine, TGF-β, acts as a central mediator for the wound-healing process^19^. Our current study suggested that TGF-β secreted by fibroblasts and inflammatory cells was responsible for inducing abnormally high levels of cell proliferation, fibroblast recruitment, and ECM synthesis. In this study, TGF-β was demonstrated to stimulate cell proliferation and myofibroblast differentiation and migration^12^. These results supported the conclusion reached by Cordeiro^20^, who suggested that TGF-β induced the proliferation and differentiation of fibroblasts, as well as ECM deposition, both in vivo and in vitro. Therefore, in this study, we used TGF-β as a positive control to mimic the microenvironment in human after filtration surgery. Our in vitro glaucoma model was established by treating cells with TGF-β.

Our microarray data showed that NR_003923, a novel lncRNA located at ch13: 51568646-51640293, with total length of 3025 bp, was transcribed from its parent gene, *GUCY1B2*, which also codes for members of the GC protein family. GCs are nitric oxide sensors which regulate IOP, and play crucial roles in glaucoma^17,21,22^. In human glioma cell lines, an overproduction of cyclic GMP (cGMP) results in elevated levels of GUCY1A1 and GUCY1B3 expression. Transfection of glioma cell lines with an antisense sequence for GUCY1A1 and GUCY1B3 significantly reduces the abundance of cGMP and expression of VEGF^23^. In addition, GCs regulate IOP and the pathophysiology of glaucoma via activation of cGMP^17^. To date, little research has focused on the biological functions of lncRNA NR_003923. In this study, our microarray data showed that NR_003923 expression was significantly upregulated in glaucoma tissues when compared with normal tissues. We further confirmed that suppression of NR_003923 significantly reduced TGF-induced cell proliferation and migration of HTFs.

Accumulating evidence suggests that lncRNAs play a key role in mediating the physiological and pathological alterations that occur in glaucoma patients. LncRNAs mediate the phenotype of fibroblasts by regulating other factors. MEG3 is a regulatory molecule that participates in the TGF-β pathway^24^ and negatively regulates the viability of HTFs by binding to nuclear factor Nrf2, which is a transcription factor associated with fibroblast proliferation and oxidative stress. In addition, lncRNA GAS5 has been demonstrated to be a key regulator in glaucoma, because of its ability to regulate the TGF-β/Smads pathway^25^. In our present study, NR_003923 was confirmed to alter the phenotypes of HTFs by sponging miR-760 and miR-215-3p, which are two miRNAs known to inhibit cell proliferation and migration^26,27^.

As a receptor for IL22, IL22RA1 has been demonstrated to be involved in inflammatory diseases^19^. A previous study showed that IL-22R was activated by IL-22 produced in the microenvironment of human glioblastoma cells, and extends the survival times of cells. Moreover, Khosravi et al. suggested that IL22 promoted cell proliferation by creating apro-inflammatory microenvironment. Researchers have suggested that the IL22/IL22RA pathway mediated fibrosis in the liver^20^. In the present study, we found that the expression of IL22RA1 was significantly upregulated in glaucoma tissues compared with normal tissues. Furthermore, IL22RA1 was confirmed to be a target of both miR-760 and miR-215-3p by the luciferase assay. Thus, we suggested that inhibition of lncRNA NR_003923 suppressed TGF-induced HTFs proliferation and migration through the miR-760/miR-215-3p-IL22RA1 pathway.

Recent studies have demonstrated that autophagy plays essential roles in maintaining the physiological/pathophysiological status of cells, including their proliferation^28^, migration^29^, and fibrosis process^30^ in various diseases. Patients with exfoliation syndrome, a systemic disorder of extracellular elastic matrices that causes a distinct form of human glaucoma, display a significant accumulation of the autophagosome marker LC3BII, when compared with control subjects^31^. TGF-β is an important regulator of fibrogenesis in different disease, and takes part in autophagy^32^. Glaucoma-related genes which involve autophagy contribute to the disease pahtogenesis^33^. Here, we shown that autophagy induced by TGF-β was confirmed to be a stimulatory signal that affected the proliferation, migration, and fibrosis transition of HTFs via its effects on the NR_003923/miR-760/miR-215-3p/IL22RA1 pathway.

In conclusion, we performed a microarray analysis of lncRNAs and mRNAs found in clinical tissues. LncRNA NR_003923 was selected for further investigation as it was upregulated in glaucoma tissues and was known to have functions in HTFs. Our results indicated that NR_003923 acted as a sponge that regulated the levels of miR-760 and miR-215-3p, which targeted IL22RA1, and thereby effects the proliferation and migration of HTFs, and also the fibrosis transition in HTFs. This research provided the first evidence for ceRNA involvement in glaucoma, and also provided information that was valuable for future investigations and clinical instruction.

## Materials and methods

### Patients and tissue collection

From January 2017 to March 2018, six pairs of human fascia tissue and pair-matched human fascia tissue (3 mm from the limbus conjunctivae) were collected from glaucoma patients at the Second Xiangya Hospital of Central South University. The tissue samples were snap-frozen in liquid nitrogen and then stored at −80 °C for further use. All procedures conducted in this study were performed in compliance with rules described in the Declaration of Helsinki. All patients provided their written were informed consent prior to surgery and tissue collection. The study protocol was approved by the Human Ethics Committee of the Second Xiangya Hospital of Central South University (Changsha, China).

### Cell culture

HTFs were isolated from three Tenon tissues in three different patients as previously described. The HTFs were cultured in Dulbecco’s Modified Eagle Medium supplemented with 10% (v/v) fetal bovine serum (Hyclone Laboratories, Logan, UT, USA) and 1% streptomycin-penicillin (Gibco, USA) in a humidified atmosphere at 37 °C with 5% CO_2_. HTFs at passages 4–6 were used for further experiments. Prior to each experiment, the cells were allowed to reach a subconfluent status (~80% confluence); after which, they were cultured in serum-free medium for 24 h.

### Microarray

Arraystar human LncRNA microarrays were performed to detect the relative levels of lncRNA and mRNA expression. The total RNA was extracted from 6 pairs of clinical tissues using TRIzol reagent (Invitrogen Life Technologies, Carlsbad, CA, USA), and microarray hybridization was performed according to the manufacturer’s instructions. A 1 μg sample of total RNA from each tissue specimen was amplified and transcribed into fluorescent cRNA using an Arraystar RNA Flash labeling kit (Arraystar, Rockville, MD, USA) as described in the manufacturer’s protocol. Next, Arraystar V4.0 was used to hybridize the fluorescent cRNA with the lncRNA arrays. After washing, the hybridized arrays were scanned with an Agilent DNA Microarray Scanner, and the resultant data was collected by Agilent Feature Extraction software (V11.0.1.1; Agilent Technologies, Santa Clara, CA, USA). After a data normalization process, quantification was performed using Agilent GeneSpring GX software (V12.1, Agilent Technologies). Low intensity RNAs were filtered out, and lncRNAs and mRNAs that were flagged as “Present” or “Marginal” in at least one out of six samples were chosen for further analysis. The threshold settings for a significantly expressed lncRNA or mRNA were a *p*-value < 0.05 and z |log2(FC)| ≥ 1.

### Recombinant plasmids and transfection

Full length NR_003923 was obtained by the 5′-3′-rapid amplification of cDNA ends. Point mutations in the miR-760 and miR-215-3p response elements were introduced (miR-760 seed sequence-binding site 5′-CAGAGCC-3′ changed to 5′-TCATCG-3′; miR-215-3p seed sequence-binding site 5′-ATGACAG-3′ changed to 5′-CATCTGT-3′) by using mismatched primers; (mismatched primers for the miR-760-binding site: F, 5′-CAGTCCCACAGCCTATCATCGATTGAAAAATCAAGGG-3′, R, 5′-CCCTTGATTTTTCAATCGATGATAGGCTGTGGGACTG-3′; for miR-215-3p: F, 5′-TGAAGATGTCTGGCTCATCTGTGATGCTACGGACACT-3′, R, 5′-AGTGTCCGTAGCATCACAGATGAGCCAGACATCTTCA-3′). The NR_003923 WT and MUT were then subcloned into the *XhoI* and *NotI* sites of thepsi-CHECK2 vector (Promega, Madison, WI, USA). Recombinant psi-CHECK2 plasmids containing the IL22RA1 3′UTR were constructed as NR_003923 WT and NR-003923-MUT, respectively, by using the methods described above.

The interactions of miRNA-mRNA pairs were predicted by using Target Human Scan software (Version 7.2; http://www.targetscan.org/vert_72/) and DIANA database, and lncRNA-miRNA interactions were predicted by Kangchen Bio-Tech (Shanghai, China) and lncRNA downstream target database of StarBase v2.0,25.

The nucleotide sequences that encoded for IL22RA1 were amplified via PCR performed with the following primers: (F, 5′-GGGGTACCATGAGGACGCTGCTGACCATCTTGA-3′; R, 5′-CCGCTCGAGTCAGGACTCCCACTGCACAGTCAGG-3′). Those sequences were then subcloned into the *Kpn*I and *Xho*I sites of a pcDNA 3.0 vector. An empty pcDNA 3.0 vector was used as a control.

siRNA, a miRNA inhibitor, and mimics were obtained from GenePharma (Suzhou, China). HTFs were seeded into the wells of sixwell plates and cultured to ~80% confluence. Transfections were performed using Lipofectamine 2000 (Invitrogen) as described in the manufacturer’s instructions. For knockdown of NR_003923, firstly we confirmed two effective siRNAs by qRT-PCR (Fig. S2). Then, two different siRNAs (siRNA-1 and siRNA-2) were mixed and transfected. siRNA-1: 5′-GCAGGAACATGTATGGATT-3′; siRNA-2: 5′-CCAAGAGGCCTGCAACATT-3′; siRNA-3: 5′-GGAAGTGACGAATCCTGTT-3′.

### Wound-healing assay

HTFs were seeded into the wells of a 6-well plate (~5 × 10^6^ cells per well) and cultured to 90% confluence; after which, they were transferred and maintained in serum-free medium for 4 h. A 1 mL micropipette tip was used to gently create a single scratch (~0.5 cm in length) in the center of a cell monolayer, and the detached cells were washed away with PBS. The scratched plates were transferred into an incubator and cultured for 48 h; after which, bright field images of the scratched areas were obtained and analyzed using Image Plus Pro to evaluate cell motility.

### Western blotting

Western blot assays were performed as previously described^34^. Briefly, the total cellular proteins were extracted by using reagents in a Radio Immunoprecipitation Assay kit (Beyotime, Shanghai, China). The extracted proteins were then separated by SDS-PAGE, and the individual protein bands were transferred onto a PVDF membrane, which was subsequently blocked with 5% skim milk. The membrane was then incubated with primary antibodies against E-cadherin (1:800, ab76055, Abcam, Cambridge, MA, USA), FN (1:5000, ab45688, Abcam, USA), β-catenin (1:8000, ab32572, Abcam, USA), α-SMA (1:1000, ab28052, Abcam, USA), LC3B (1 μg/mL, ab48394, Abcam, USA), p62 (1:2000, ab155686, Abcam, USA), or the internal reference GAPDH (1:20000, ab128915, Abcam, USA). Next the membrane was incubated with HRP-conjugated goat anti-mouse or goat anti-rabbit secondary antibodies. An enhanced chemiluminescence detection system was used to detect areas of luminescence, and the relative-staining intensity of each protein band was quantitated using Image Plus Pro software (Ver. 6.0, Media Cybernetics, Inc., Rockville, MD, USA).

### Immunofluorescent assay

HTFs were seeded onto coverslips that had been placed into the wells of a sixwell plate (5 × 10^5^cells/well). Next, the cells were fixed with 4% paraformaldehyde, permeabilized with 0.3% Triton X-100 for 6 min, and then blocked with 2% normal goat serum (Cat. C-0005; HAORANBIO, China). The cells were then incubated overnight at 4 °C with primary antibodies against E-cadherin (ab76055, 1:100), FN (ab32419, 1:100), β-catenin (ab16051, 1:200), α-SMA(ab108531, 1:200), LC3B (ab192890, 1:500), and p62 (EPR18351, 1:200). After incubation with the primary antibodies, the cells were incubated with a FITC-labeled secondary antibody for 1 h at 25 °C, and their nuclei were stained with DAPI. Finally, the stained cells on the coverslips were observed under a laser-scanning confocal microscope.

### Cell viability assay

Cell viability was measured using a Cell Counting Kit-8 (CCK8) (Lianke Bio, Hangzhou, Zhejiang) according to manufacturer’s instructions. In brief, cells at a density of 1 × 10^4^ cells/well were seeded in a 96-well plate in triplicate. After the cells had received their specified treatment, a CCK8 working reagent was gently added to each well, and the plates were placed in a 37 °C incubator for 2 h. Finally, the absorbance of each well at 450 nm was measured with a microplate reader (Multiskan FC, Thermo Fisher Scientific, Waltham, MA, USA).

### Real-time PCR

The total RNA and miRNA in cells or tissues was isolated using TRIzol reagent (Takara, Japan) according to the manufacturer’s instructions. Reverse transcription into cDNA was performed using a DNA synthesis kit (Cat.K1622, Permentas, USA). PCR reactions were performed using Master Mix (SYBR Green Kit, Cat. DBI-2043, DBI Bioscience, Newark, DE, USA) according to the manufacturer’s instructions. The quantitative values for mRNA and miRNA data were normalized to the values for GAPDH mRNA and U6 snRNA, respectively. Data were analyzed using the 2^−ΔΔCt^ method.

### Statistical analyses

All experiments were performed in triplicate, and the data were analyzed using IBM SPSS Statistics for Window, Version 22.0 (IBM Corp, Armonk, NY, USA). Results are presented as the mean ± SD. Differences between groups were analyzed by using the *t*-test or ANOVA followed by the Tukey test. *P*-values < 0.05 were regarded as statistically significant.

## Supplementary information

Supplementary results.

Figure S1

Figure S2
