# Supplementary material for: LncRNA NR_003923 promotes cell proliferation, migration, fibrosis, and autophagy via the miR-760/miR-215-3p/IL22RA1 axis in human Tenon’s capsule fibroblasts
Source: Cell Death Dis. 2019 Aug 7;10(8):594. doi: 10.1038/s41419-019-1829-1 (PMC6685939; doi:10.1038/s41419-019-1829-1)
Supplement: Supplementary file 1 — Supplementary results. [file 41419_2019_1829_MOESM1_ESM.docx]

**Supplementary Figure Legends**

**Figure S1**. TGF-β promoted the proliferation, migration, and fibrosis transition of Tenon’s capsule fibroblasts. **A.** Cell proliferation was activated by treatment with 1, 2, or 4 ng/mL TGF-β. **B and C.** TGF-β significantly increased cell migration. **D, E, and F.** E-cadherin and β-catenin mRNA (**D**) and protein levels (**E and F**) were suppressed by TGF-β, while α-SMA and FN (fibronectin) levels were enhanced by TGF-β. *p < 0.05, **p < 0.01 vs 0 ng/mL.

**Figure S2.** qRT-PCR analyzed the expression of NR_003923 in HTFs after transfected with siRNAs.
