# Supplementary figures and images for: LncRNA NR_003923 promotes cell proliferation, migration, fibrosis, and autophagy via the miR-760/miR-215-3p/IL22RA1 axis in human Tenon’s capsule fibroblasts
Source: Cell Death Dis. 2019 Aug 7;10(8):594. doi: 10.1038/s41419-019-1829-1 (PMC6685939; doi:10.1038/s41419-019-1829-1)

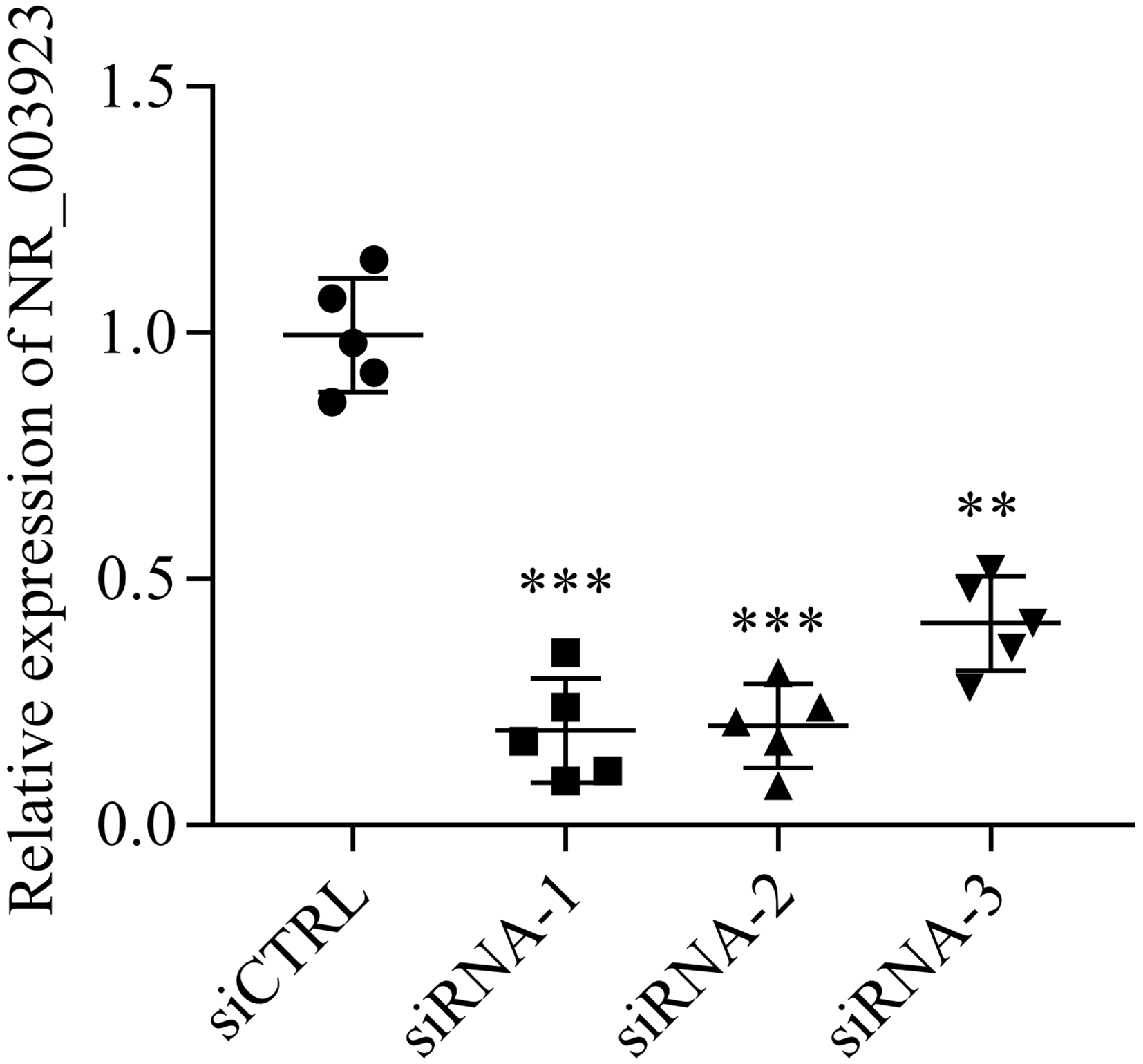

Supplement: Supplementary file 3 — Figure S2 [file 41419_2019_1829_MOESM3_ESM.tif]
